# Effects of Litter Manipulation on Litter Decomposition in a Successional Gradients of Tropical Forests in Southern China

**DOI:** 10.1371/journal.pone.0099018

**Published:** 2014-06-05

**Authors:** Hao Chen, Geshere A. Gurmesa, Lei Liu, Tao Zhang, Shenglei Fu, Zhanfeng Liu, Shaofeng Dong, Chuan Ma, Jiangming Mo

**Affiliations:** 1 Key Laboratory of Vegetation Restoration and Management of Degraded Ecosystems, South China Botanical Garden, Chinese Academy of Sciences, Guangzhou, China; 2 Department of Geosciences and Natural Resource Management, University of Copenhagen, Copenhagen, Denmark; 3 State Key Laboratory of Urban and Regional Ecology, Research Center for Eco-Environmental Sciences, Chinese Academy of Sciences, Beijing, China; 4 Institute of Tropical Pratacultural Science, Zhanjiang Normal University, Zhanjiang, China; 5 University of Chinese Academy of Sciences, Beijing, China; 6 Sino-Danish Center for Education and Research, Aarhus, Denmark; DOE Pacific Northwest National Laboratory, United States of America

## Abstract

Global changes such as increasing CO_2_, rising temperature, and land-use change are likely to drive shifts in litter inputs to forest floors, but the effects of such changes on litter decomposition remain largely unknown. We initiated a litter manipulation experiment to test the response of litter decomposition to litter removal/addition in three successional forests in southern China, namely masson pine forest (MPF), mixed coniferous and broadleaved forest (MF) and monsoon evergreen broadleaved forest (MEBF). Results showed that litter removal decreased litter decomposition rates by 27%, 10% and 8% and litter addition increased litter decomposition rates by 55%, 36% and 14% in MEBF, MF and MPF, respectively. The magnitudes of changes in litter decomposition were more significant in MEBF forest and less significant in MF, but not significant in MPF. Our results suggest that change in litter quantity can affect litter decomposition, and this impact may become stronger with forest succession in tropical forest ecosystem.

## Introduction

Litter decomposition is a key process that regulates nutrient recycling in ecosystems, influences net ecosystem carbon (C) storage, and is the first step in the formation of soil humus [1]. Litter decomposition can be affected by many environmental factors including the physical environment (e.g. temperature, moisture, and soil pH), nutrients availability and activities of decomposers in the soil [2]. On the other hand, litter quantity can alter microclimate, number and dynamics of decomposer organisms and nutrient availability in forest floor and mineral soil [3]. Thus, the quantity of the litter itself has an impact on litter decomposition. It has been reported that global climate changes due to rising atmospheric CO_2_ concentration and temperature can increase net primary production (NPP) and consequently litter production in forest ecosystems [4]–[6]. In contrast, litter inputs are also likely decreased due to extensive deforestation and cultivation [7]. Therefore, evaluating the effects of these changes in litter inputs on litter decomposition is crucial for our understanding of ecosystem nutrient supplies and future global C cycle.

Despite the substantial number of studies on decomposition in a wide range of ecosystems, the influence of a sustained change in litter quantity on litter decomposition has not been well addressed [3]. Existing evidence on the effects of litter quantity on the leaf litter decomposition is indirect, and the results are inconsistent and incomparable. For example, comparison of litter decomposition before and after hurricanes showed that the large inputs of litter following hurricanes would cause accelerated or slower decomposition rate [8], [9]. Similarly, results from the labile-C (glucose, cellulose, or root exudates) addition experiments have shown that labile-C addition could have both positive and negative effect on leaf litter decomposition [10]–[13]. Moreover, potentially decreased litter quantity after understory removal also showed both decreasing [14], [15] and increasing [16] effects on litter decomposition.

Compared to the methods mentioned above, litter manipulation (removal or addition) experiment is considered to be a direct way for studying effects of litter quantity on ecosystem processes [17]. Using this method, recent studies have reported that increased litter input would accelerate soil C release and decreased soil C content in tropical forests due to the “priming effect” [18]–[21]. However, most of these studies assessed the effect of litter quantity on the decomposition of soil organic matter and only few studies addressed the effect on decomposition of fresh leaf litter. In addition, more studies focused on the effect of litter removal [22], [23] and to our knowledge only one studied effects of litter addition on litter decomposition in forest ecosystem [3], highlighting the need for more complete comparative study on effects of both litter addition and removal on leaf litter decomposition.

While estimates of decomposition after litter manipulation are commonly reported in single forest type, forests at different successional stages are likely to present distinct responses. The Dinghushan Biosphere Reserve (DHSBR) consists of three typical forest types in southern China at different forest successional stages in terms of age and exposure to human disturbances; hence it provides an excellent opportunity to study the response of litter decomposition to litter manipulation along a forest successional gradient. The three forest types are a pioneer masson pine forest (MPF), a transitional coniferous and broad-leave mixed forest (MF) and a climax monsoon evergreen broad-leaved forest (MFBF) [24]. Previous studies showed that these forests have large variation in litter production, nutrient status in plant and soil, and some other environment factors [24]–[26]. This study presents the results of litter decomposition experiment where we quantitatively measured the effects of litter removal and addition on litter decomposition in three forests in DHSBR. Our objectives were to examine the effects of litter removal and addition on litter decomposition in tropical forests and to compare these effects among forests at different successional stages. We hypothesized that (1) litter removal would decrease litter decomposition but litter addition would increase litter decomposition in each forest types due to the priming effect; and (2) response of litter decomposition to litter removal/addition would vary among the three forests.

## Materials and Methods

### Ethics Statement

No specific permits were required for the described field studies. This research station (DHSBR) belongs to South China Botanical Garden, Chinese Academy of Sciences, which also supported the study. We confirmed that the location is not privately owned. We also confirmed that the field studies did not involve endangered or protected species. Data will be made available upon request.

### Study Site

The study was conducted in Dinghushan Biosphere Reserve (DHSBR), which is in the middle part of Guangdong province, southern China (112^o^33’ E and 23^o^10’ N). Average annual precipitation is 1927 mm, with 75% occurring from March to August and only 6% from December to February [27]. Mean annual relative humidity is 80%. Mean annual temperature is 21.0°C with the lowest and highest average temperature being in January (12.6°C) and July (28.0°C) [27]. Soil type is lateritic red earth (Ultisols in the USDA soil taxonomy or Acrisols in the FAO soil classification) formed from sandstone [28].

There are three main forest types in the reserve; a pioneer masson pine forest (hereafter named as MPF), a coniferous and broad-leave mixed forest (hereafter named as MF) and a monsoon evergreen broad-leaved forest (hereafter named as MEBF) [24]. The three forest types form distinct successional gradients [24], [29]. The MPF belongs to the first stage of the successional processes and occurs in the transition zone (periphery) of the reserve at an elevation of about 200m. *Pinus massoniana* Lamb is the dominant species in MPF (Table S1), which was planted in the 1930s. Top soil (0–30 cm) texture in MPF is medium gravel-medium loam (Ultisols in the USDA soil taxonomy), and the capacity of field moisture and wilting coefficient in the soil expressed as gravimetric water content are 26.0% and 10.9%, respectively [30]. The MF is distributed on areas next to the MPF and towards the core areas of the reserve at an elevation of about 200–300 m. It was developed by a gradual invasion of the originally planted MPF by some pioneer broadleaf species through natural succession [31]. The plant composition in MF has greatly been changed. Dominant tree species in the mixed forest are *Pinus massoniana* Lamb, *Schima superba* Chardn. & Champ., *Cryptocarya chinensis* Hance, *Craibiodendron kwangtungense* S. Y. Hu, *Lindera metcalfiana* Allen, and *Cryptocarya concinna* Hance (Table S1). The texture of top soil in MF is medium gravel-heavy loam with field capacity and wilting point at 25.3% and 8.2% gravimetric water content, respectively [30]. The MEBF is distributed in the core area of the reserve at an elevation varying from 250 to 300 m. It has been protected from direct human impact for more than 400 years [28]. Major species in MEBF are *Castanopsis chinensis* Hance, *Schima superba* Chardn. & Champ., *Cryptocarya chinensis* (Hance) Hemsl., *Machilus chinensis* (Champ. ex Benth.) Hemsl., *Syzygium rehderianum* Merr. & Perry in the canopy layer (Table S1) and *Calamus rhabdicladus* Burret, *Ardisia quinquegona* B1. and *Hemigramma decurrens* (Hook.) Copel in the understory layer. The texture of top soil in MEBF is light gravel-heavy loam. The water retention capacity of top soil is the highest with field moisture capacity and wilting coefficient of 34.6% and 11.4% gravimetric water content, respectively [30]. Litter layers are different among three forests. Litter layer is thin in the MEBF due to the faster decomposition rate, but thick in the MF and MPF. The three forests also vary in litter production, nutrient status in soil and plant, and some other environmental factors. See Table 1 for information on selected site characteristics of the three forest types.

**Table 1:** Comparisons of litter production, leaf litter N and P concentration and selected soil properties among MEBF, MF, and MPF.

Forest types	MEBF	MF	MPF
Litter production (Mg ha^−1^ yr^−1^)‡	8.3 (0.64)^a^	8.5 (0.62)^a^	3.3 (0.57)^b^
Leaf litter N (mg g^−1^)^†^	17.5 (0.12)^a^	15.0 (0.10)^b^	9.6 (0.07)^c^
Leaf litter total P (mg g^−1^)^†^	0.53 (0.01)^a^	0.32 (0.01)^b^	0.36 (0.01)^b^
Soil N (mg g^−1^)	1.99 (0.18)^a^	0.93 (0.08)^b^	1.15 (0.16)^b^
Soil organic matter (%)	7.3 (0.8)^a^	3.7 (0.2)^b^	5.2 (0.2)^b^
Soil C/N ratio	21.0 (0.6)^a^	23.8 (2.2)^a^	28.1 (3.9)^b^
Soil total P (mg g^−1^)	0.49 (0.03)^a^	0.38 (0.01)b	0.44 (0.01)^ab^
Soil available P (mg kg^−1^)	2.2 (0.5)^ab^	1.5 (0.5)^a^	2.9 (0.2)^b^
Soil moisture (%)	22.6 (1.1)^a^	16.4 (1.9)^b^	15.3 (1.1)^b^
Soil temperature (°C)	21.8 (0.36)^a^	22.6 (0.37)^b^	23.41 (0.39)^c^

‡ from Zhou et al. (2011); ^†^leaf litter chemical characteristics were average values of main litter species in the each forest, which were measured in 2012 in the control plots. Other values were measured in August 2007 in the control plots. Values are means, standard error in parentheses, n = 5, means not sharing the same superscript letter were statically different at *P*-value of 0.05.

### Experimental Design

In each forest, we used a randomized complete block design with five blocks (i.e., n = 5). In each block, we set up three 1 m×1 m plots to be used as a control (CT), a litter removal (L−), and a litter addition (L+) plots making up a total of 15 plots in each forest. There was at least 3 m buffer zone between two adjacent plots to avoid overlapping effects of different treatments. The litter decomposition experiment started in February, 2007, by placing litterbags on the surface of litter layer in the plots. We removed all litter every month from the L− plots and added it to the corresponding L+ plots to cover the litterbags. Litter removal from the L− plots caused low average litter input but did not cause entire absence of litter input to the plots because the plots was receiving litter in the following days of each mouth. The controls received the normal input of aboveground litter.

### Litter Collection and Initial Nutrients Analysis

Leaf litter was collected using litter traps and nylon mesh placed on the forest floor under the trees in the study sites in May and June 2006, during which litterfall is peak [32]. Leaf litter of *Schima superba* Chardn. & Champ. (SS) and *Castanopsis chinensis* Hanc (CC) was collected in MEBF and MF. Leaf litter of SS, CC and *Pinus. massoniana* Lamb (PM) was collected in MPF. These species were chosen in this study because they are the dominant tree species and contribute 40–91% the total leaf litter fall in these forests [33]. All litter was air-dried to a constant weight and six sub-samples from each kind of litter were analyzed for initial nitrogen (N) and phosphorus (P) concentrations (Table S2). N concentration was determined by the semimicro-Kjeldahl digestion method followed by the detection of ammonium with a Wescan ammonia analyzer, while total P concentration was analyzed colorimetrically after acidified ammonium persulfate digestion [34].

### Litter Decomposition Experiment

Litter decomposition was determined by placing fresh litter in mesh bags in the plots. A total of 2625 litter bags were prepared from 25×25 cm polyvinyl screen with 0.5×0.5 mm mesh in the bottom and 2×2 mm in the top. Leaf litter of each species was mixed before filling in the mesh bags. Each bag was filled with 10 g air-dried mass of litter. Only one litter type was put in each bag and the litter bags were evenly distributed among each plot. Litter bags were retrieved at about 3, 6, 9, 12, and 18 months (hereafter named as first, second, third, fourth and last sampling date, respectively) after the start of the study. Five litter bags of each species (a total of 525 bags on each collection) were randomly selected and collected from each plot at each sample date. The average of the 5 litterbags of each species per plot at each sampling date was used in the statistical calculations. After removing roots, soil and other extraneous materials, the leaf residue in each litter bag was oven dried at 45°C for 48 h and weighed. Litter from the last sampling date was measured for N and P concentrations using the method as described above.

### Soil and Microbial Sampling

Soil sampling was conducted in August 2008 (1.5 years after litter manipulation). From each plot, 5 soil cores with 2.5 cm inner diameter were collected at random points from a 0–10cm soil depth and combined to create one composite sample per plot. Changes in soil microbial biomass was studied only in MEBF using phospholipid fatty acid (PLFA) analysis as described in Bossio and Scow [35], because samples from the other two forests were polluted during transportation of the samples from fields.

### Calculations and Data Analysis

To determine litter decomposition rates we used the following decomposition model [36]: y = e ^(−*k*t)^ (exponential model), where y is the fraction of mass remaining at a specific time t (years), “e” the base of natural logarithm, *k* the decomposition coefficient (year^−1^). To determine changes in decomposition due to treatment effect, *k* value changes were given by (*k*_trt_–*k_con_*)/*k*_con_, where *k*_trt_ is the decomposition coefficient in treatments (L− or L+), *k_con_* the decomposition coefficient in control. Nutrient content was calculated by multiplying the nutrient concentration by the mass remaining. Nutrient content was then reported as a proportion of the initial leaf content [37].

One-way Analysis of Variance (ANOVA) was used to test the difference in decomposition rate (*k*) among forests or litter species in controls, and to test the difference in PLFAs among treatments in MEBF. Two-way ANOVA was used to determine the interaction effect of litter manipulation treatments and litter types on litter decomposition rate and nutrient remaining in each forest. Repeated measure ANOVA was used to determine the difference in litter mass remaining among forests, litter manipulation treatments and litter species. In all ANOVAs, the block was included as random factors. In addition, data was log transformed to fulfill the requirements of normality and homogeneity of variance. All analyses were conducted using SAS software (SAS Institute Inc., Cary NC, USA). Statistically significant differences were set with *P* values <0.05 unless otherwise stated. Mean values ± standard errors were reported in the text.

## Results

### Litter Decomposition Rate in Control Plots

**Figure 1:**
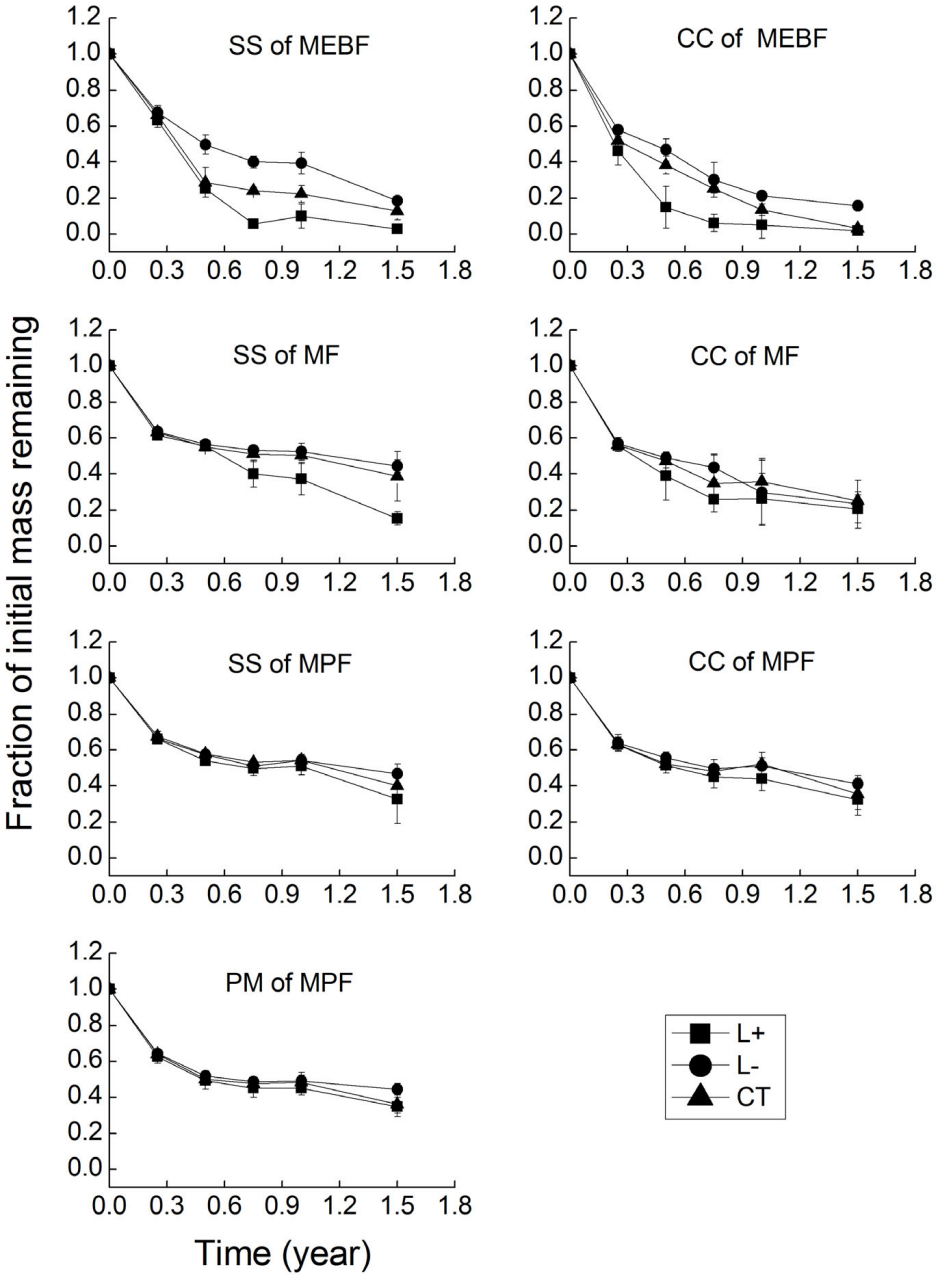
Mass loss of decomposing leaf litter of three dominant tree species in various litter manipulation treatments in the MEBF, MF, and MPF.

The leaf litter decomposition rates in control plots varied among forest types and tree species (Fig. 1, Table 2). Leaf litter decomposition rate (i.e., *k* value) was significantly higher in MEBF than in MF and MPF for all possible species comparison. However, no significant difference in litter decomposition rate was found between MF and MPF for all species. Average litter decomposition rates (*k*) were 1.97, 0.92, and 0.73 for MEBF, MF, and MPF, respectively. When compared within forest type, litter decomposition rate was significantly lower in CC leaf litter than in SS in the MEBF and MF (Table 2, *P* = 0.037 and 0.021, respectively). However, there was no significant difference among the selected three species in the MPF (Table 2, *P* = 0.261).

**Table 2:** Comparisons of decomposition rates (*k* values) between treatments and litter species in each forest.

Species	Treatments	MEBF		MF		MPF	
		*K*	*R^2^*	*K*	*R^2^*	*k*	*R^2^*
SS	CT	1.74 (0.36)^a^	0.84 (0.04)	0.80 (0.05)^a^	0.60 (0.09)	0.68 (0.04)^a^	0.71(0.06)
	L–	1.23 (0.19)^b^	0.86 (0.04)	0.66 (0.02)^b^	0.57 (0.07)	0.64 (0.02)^a^	0.54 (0.09)
	L+	2.80 (0.24)^c^	0.86 (0.06)	1.13 (0.05)^c^	0.81(0.04)	0.84 (0.09)^a^	0.69 (0.07)
CC	CT	2.19 (0.06)^c^	0.75 (0.06)	1.04 (0.06)^cd^	0.68 (0.10)	0.78 (0.05)^a^	0.62 (0.09)
	L–	1.63 (0.29)^a^	0.80 (0.04)	1.01 (0.05)^cd^	0.77 (0.07)	0.72 (0.04)^a^	0.60 (0.06)
	L+	3.30 (0.25)^d^	0.84 (0.04)	1.35 (0.12)^d^	0.85 (0.09)	0.89 (0.08)^a^	0.75 (0.07)
PM	CT	–		–		0.80 (0.03)^a^	0.64 (0.04)
	L–	–		–		0.71 (0.02)^a^	0.51 (0.06)
	L+	–		–		0.84 (0.05)^a^	0.64 (0.09)

Notes: *k* values and coefficients of determination (*R^2^*) are based on a single negative exponential model. Two-way ANOVA with SNK test was used in each forest respectively to determine the effect of litter treatments and litter types on *k* values. Values are means, standard error in parentheses, n = 5. Under each forest type and for each variable column, means not sharing the same superscript letter were statically different at *P*-value of 0.05.

### Effects of Litter Manipulation on Litter Decomposition

Responses of litter decomposition rates to litter removal/addition varied with forest types (Table 2). In MEBF, litter removal significantly decreased litter decomposition rates for both SS and CC (*P* = 0.045 and 0.030, respectively). Repeated measure ANOVA with Turkey’ HSD test showed that mass remaining was significantly higher in L− than in control plots in the second, third, fourth and fifth sampling dates for SS leaf litter. For CC leaf litter, significant difference in litter decomposition between L− and control plots was observed only after the last sampling date (Fig. 1). In contrast, litter addition significantly increased litter decomposition rate for both SS and CC (*P* = 0.018 and 0.005, respectively). However, such a response of leaf litter decomposition rates to litter addition showed temporal variation between the two species. For SS, significant difference between control and L+ plots was found in the second, third, and fourth sampling dates, whereas the reported response for CC was observed in the third and fourth sampling dates (Fig. 1).

In MF, SS and CC leaf litter showed similar response to litter addition. Litter decomposition rate increased significantly after litter addition for both species (Table 2, *P* = 0.019 and 0.012 for SS and CC, respectively). However, the effect of litter removal on litter decomposition varied between the two species (Table 2). Litter removal significantly decreased litter decomposition for SS (*P* = 0.033), but not for CC (*P* = 0.842). Repeated measure ANOVA with Turkey’ HSD test indicated that litter addition significantly increased decomposition of SS leaf litter in the third, fourth and fifth sampling dates, and significantly increased decomposition of CC leaf litter in the second and third sampling dates (Fig. 1). Litter removal significantly decreased SS leaf litter decomposition in the last sampling date, but no significant change in leaf litter decomposition was observed for CC leaf litter decomposition at all sampling dates (Fig. 1).

In MPF, decomposition rate increased slightly with litter addition and decreased slightly with litter removal for all species (Table 2), but one-way ANOVA showed that this increase/decrease was not significant among treatments for all species. Repeated measure ANOVA also showed no significant difference in mass remaining among treatments over the entire study period for all species. There are no significant interaction effects of litter species and treatments on litter decomposition rate in all the three forests (*P*>0.05 for all).

**Figure 2:**
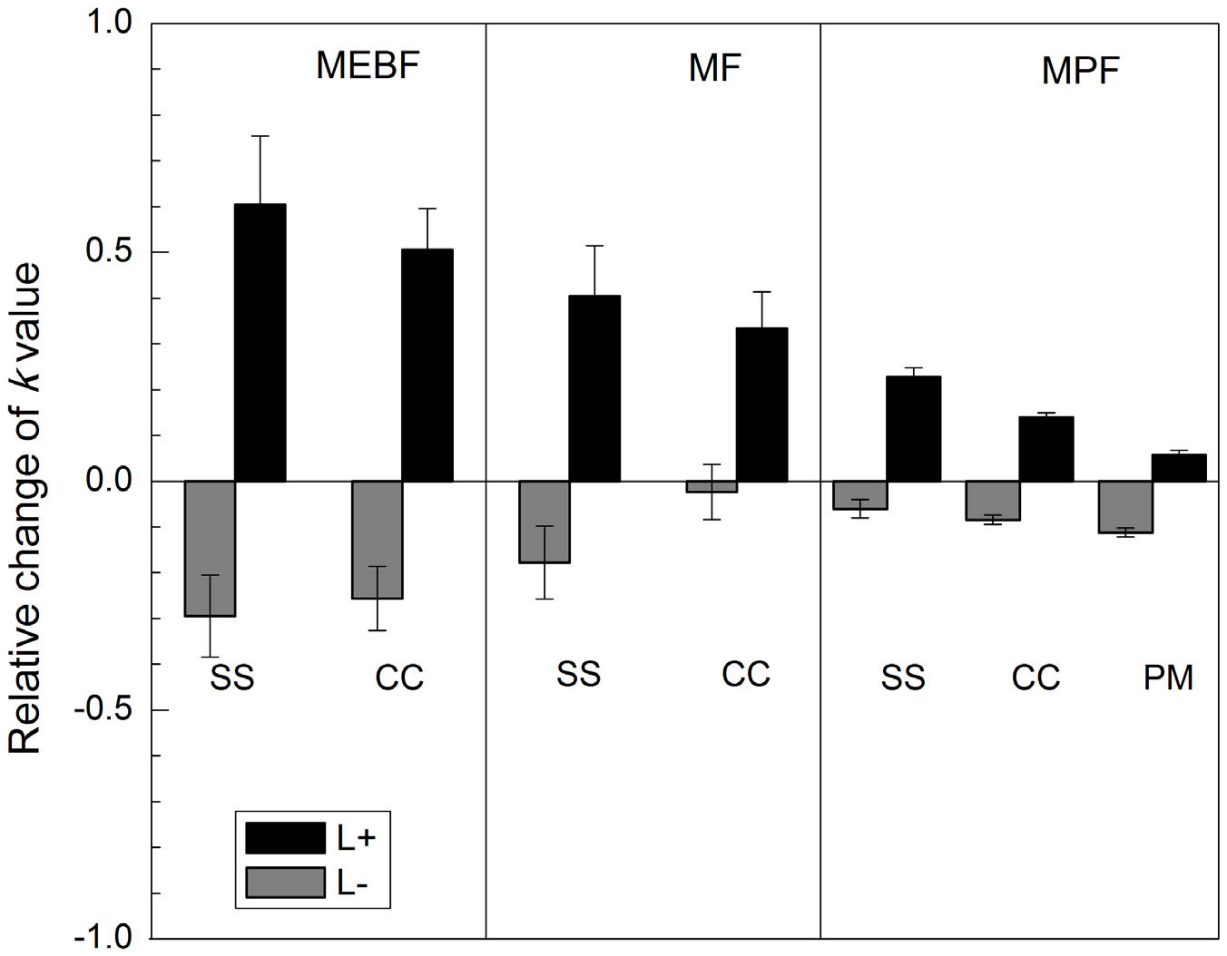
Comparisons of relative change of *k* value between different litter manipulation treatments, litter species and forest types.

The relative change in *k* values in response to the treatments decreased in the order: MEBF>MF>MPF (Fig. 2). Average decrease in litter decomposition rate as a result of litter removal was 27% in the MEBF, which was significantly higher than the respective values in MF (10%) and MPF (8%) (*P*<0.001). On the other hand, litter addition increased leaf litter decomposition by 55% in the MEBF, which is significantly larger than the average percent increase in leaf litter decomposition rates in MF (36%) and MPF (14%) (*P*<0.001).

### Litter Nutrient Remaining

**Figure 3:**
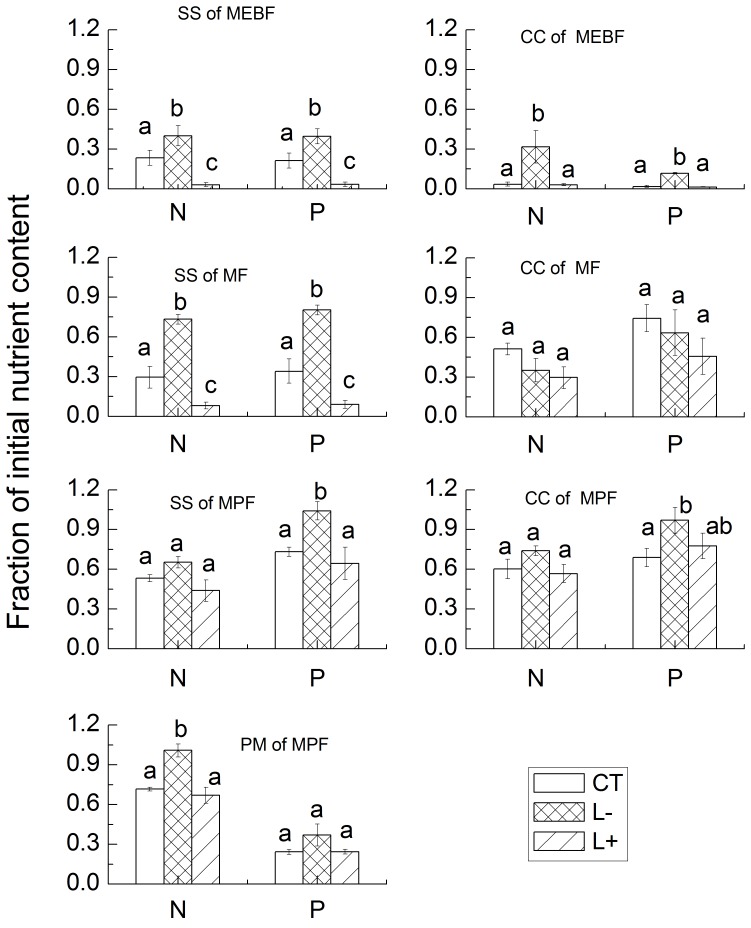
Nutrients remaining in the last stage of the decomposition. Values are means, standard error in parentheses, n = 5, means not sharing the same superscript letter were statically different at *P*-value of 0.05.

Nutrient (N and P) remaining in all leaf litter types was measured after the last sampling date and it varied depending on forests and species (Fig. 3). In MEBF, litter removal significantly increased N and P remaining in decomposing leaf litter for both SS and CC. In contrast, litter addition decreased N and P remaining in both species leaf litter, but the decrease in N and P remaining was significant only for SS leaf litter. In MF, litter removal significantly increased N and P remaining and litter addition significantly decreased N and P remaining in SS leaf litter. However, none of the treatments caused significant changes in N and P remaining in decomposing CC leaf litter when compared to the control plots, but both litter removal and litter addition tended to decrease N and P remaining in the CC leaf litter (all *P*>0.05). In MPF, litter removal tended to increase N and P remaining and litter addition tended to decrease N and P remaining in all species, but the magnitude of these changes varied among the treatments and species. For example, litter removal significantly increased N remaining in PM (*P* = 0.005), P remaining in SS and CC (*P* = 0.024 and *P* = 0.033, respectively).

### Soil Microbial Biomass

**Figure 4:**
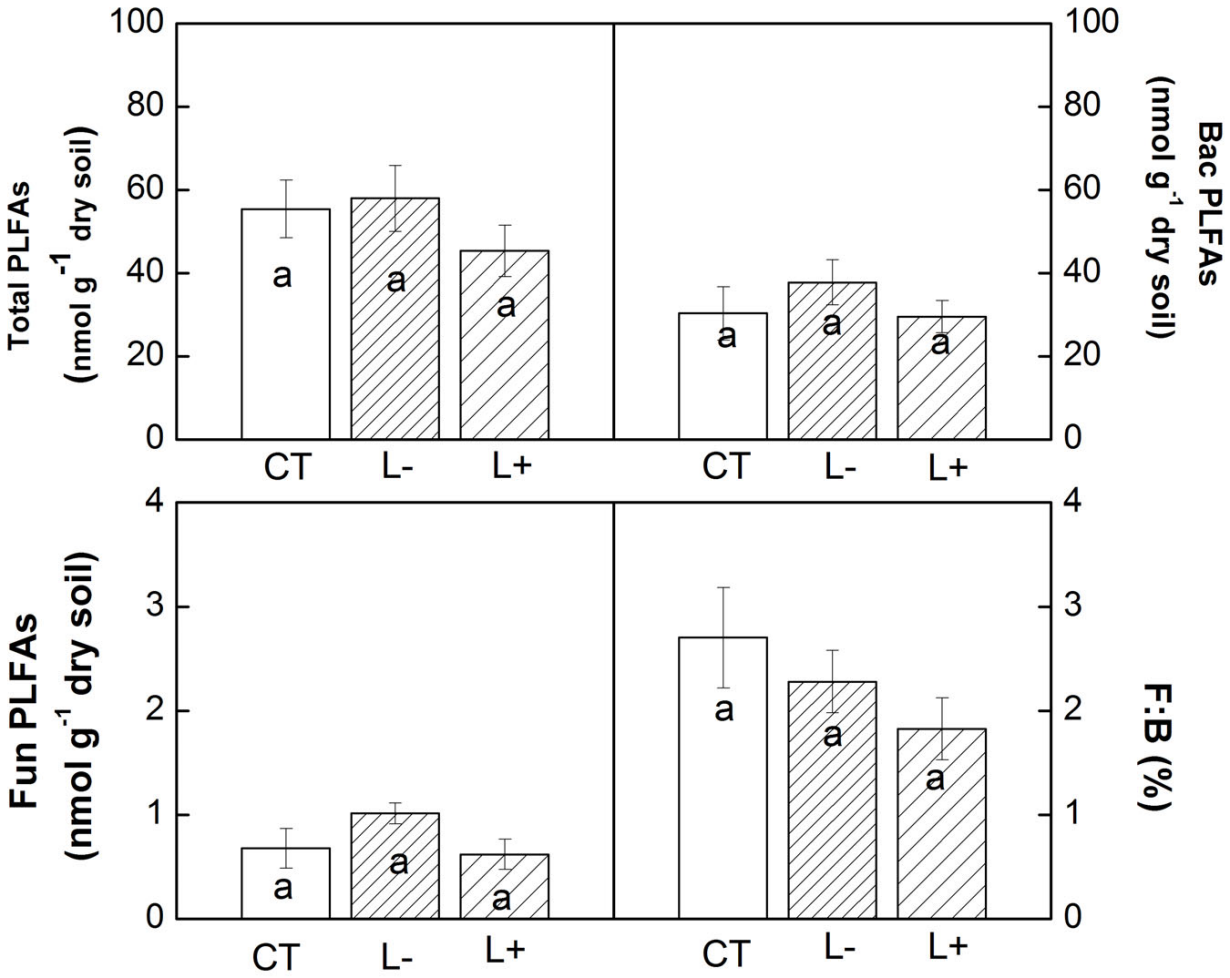
Comparisons of soil microbial PLFAs between treatments in the MEBF. Data from August 2008. Bac PLFAs: Bacterial PLFAs; Fun PLFAs: Fungal PLFAs; F:B (%): the percentage of fungal to bacterial PLFAs. Values are means, standard error in parentheses, n = 5, means sharing the same superscript letter were not statically different (*P*-value ≥0.05).

Litter manipulation had no significant effect on soil microbial biomass in the MEBF (Fig. 4, *P* = 0.074). The average total PLFAs were 55.40 (6.91), 57.94 (7.9), and 45.34 (6.1) nmol g^−1^ in control, L−, and L+ plots, respectively. Similarly, there was no significant difference among litter manipulation treatments for the Bac PLFAs, Fun PLFAs, and F:B (Fig. 4).

## Discussion

In the present study, decomposition rates (*k* values) of leaf litter observed in the controls (ranging from 0.68 to 2.19) were similar to those found in subtropical forests of south China [33], [38]–[39] and other subtropical/tropical forests [40]–[42], but were slightly lower than that of some other tropical forests with high rainfall [43]. However, the *k* values in our study were higher than those reported from temperate forests [44], [45], indicating higher decomposition rate in these humid sub-tropical forests of southern china. In addition, decomposition rate increased with forest succession: MEBF>MF>MPF, which was in agreement with results reported by previous studies in adjacent forests [33], [38].

Compared to control plots which received normal litter input, litter removal significantly decreased litter decomposition in the MEBF. Similar effect of litter removal on litter decomposition was reported by a study in an old-growth forest in Panama [3]. Based on available evidence from literature, there are several possible mechanisms that could explain our observation. Litter removal changes the microclimate (e.g. moisture and temperature) in the forest floor [46], [47], causes direct nutrient losses and changes in soil physical and chemical characteristics [48], [49], and decreases decomposer biomass and activity [3], [50]. Even though we did not measure plot microclimate, previous studies have reported that litter removal usually affects soil microclimate, mainly decreasing moisture and greater fluctuations in temperature in forest floor [46], [47]. On the other hand, a previous study observed in adjacent forests showed that litter removal had no significant effect on the soil temperature in all the three forests, and significantly decreased soil moisture only in MF [26]. However, the soil temperature and moisture were measured in mineral soil at 5 cm depth [26]. We expect more changes in forest floor microclimate following the litter manipulation, but such data are missing in our study.

Decreased nutrient supply for decomposer microbial communities after litter removal is another likely explanation for the slower decomposition in the L− plots. In this study, litter was removed once a month in L− plots, which could remove 44–73% of litter nutrient input [49]. This decreased nutrient supply might decrease microbial activities in the L− plots. We measured the litter nutrients remaining in the last sampling date and found that the content of N and P increased in L− plots compared to those in controls for all litter. This might indicate that more N and P were immobilized in L− plots, possibly because the microbes need more N and P in L− treatment due to reduced litter input of these nutrients making the microbes nutrient limited as suggested by [36].

However, we did not observe significant and clear changes in soil microbial biomass and microbial community as indicated by PLFA result (Fig. 4). Similar results were also reported from temperate forests, where change in the soil microbial biomass or activity in litter removal treatment were not significant [51]. It has been suggested that absence of expected increase in soil microbial biomass could be due to soil pools buffering the effect of litter manipulation from aboveground [51], [52]. However, our data on microbial biomass included only mineral soil because clear forest floor layer in MEBF is usually absent due to fast turn-over of organic materials. Even though data on the forest floor is not available in this study, previous study reported that litter removal decreased decomposers biomass more in forest floor than in mineral soil [3]. The study also showed that litter removal reduced the abundance of meso-arthropods in forest floor because meso- and micro-arthropods, which play significant role in decomposing organic materials, mostly inhabit top forest floor layer. Though our data only for mineral soil might not be very conclusive especially when data is absent for the more active and dynamic litter layer, it showed that microbial activities might not be affected by short term litter manipulation. However, more comprehensive studies including response of microbial dynamics to litter manipulation both in mineral soil and forest floor are needed for further understanding of the subject.

By contrast, litter addition significantly accelerated the decomposition of leaf litter in the MEBF. Our result is inconsistent with results reported by Sayer *et al.* [3], which showed that litter addition did not affect leaf litter decomposition but significantly increased wood litter decomposition. However, our result partially supports the “priming effect” hypothesis which suggests that the addition of fresh organic matter (leaf litter) can stimulate decomposition of the organic matter [53], [54]. The reason for this response is not clear in this study. We observed decreased N and P reaming in L+ plots compared to control plots indicating that increased nutrient availability from the additional litter could stimulate the microbe to release litter nutrients, which partially contribute to increased decomposition of leaf litter. In addition, we did not find significant changes in soil microbial biomass and microbial community after the litter addition (Fig. 4), but we still cannot rule out the possibility of the change of microbial biomass and microbial communities in litter layer. For example, Sayer *et al.* [3] found increased abundance of meso-arthropode in forest floor after doubling litter input resulting in increased mass loss of leaf litter. However, we could not attribute our observation to an increase in meso-arthropodes because the size of the mesh we used (0.5mm×0.5 mm) might have reduced the contribution macro-decomposer communities despite possible increases in their abundance. Comparative studies of effects of litter manipulation on litter decomposition with different mesh sizes might provide better understanding on possible confounding effects of mesh size in litter decomposition studies.

The effect of litter manipulation experiment on leaf-litter decomposition in our study varied significantly among the three forest types and exhibited clear pattern with successional gradients. The effect was highest in the climax MEBF and lowest in the MPF whereas MF showed intermediate response (Fig. 2). The reasons for this response pattern are currently not clear. However, several explanations could be suggested. Litter production was found to differ among these three forests, amounting 83, 85 and 33 Mg ha^−1^ yr^−1^ in MEBF, MF, and MPF, respectively (Table 1). Similarly, nutrient concentration and quality of leaf (C/N ratio, lignin content etc.) differs among the three forests. Foliar N and P concentrations in MF and MPF were significantly lower than in MEBF (Table 1). This suggests that more C and other nutrients in MEBF would be removed or input after litter manipulation compared to those in MF and MPF, and thus resulting in more significant response in MEBF. Another possible explanation could be different responses in soil microclimate after litter treatments among the forests, especially for soil moisture. Soil moisture is a more important factor than soil temperature for the fastest decomposition rate in the MEBF because Table 1 showed that the MEBF had the highest moisture and the lowest temperature in soil than other two forests (Table 1). Because of this higher initial moisture, we believe that the thorough litter removal/addition may change the soil moisture of the MEBF in a larger extent compared to other forests with lower initial moisture. In addition, for the MF and MPF, the long-term history of relatively more exposure to human disturbance, lower litter production and less canopy cover also might have reduced the responses to our short-term litter manipulation experiment.

## Conclusion

Our results showed that litter removal decreased litter decomposition and litter addition increased litter decomposition in three tropical forests at different successional stages. However, the effect was highest in the climax MEBF and lowest in the MPF whereas MF showed intermediate response, suggesting that the change in litter quantity can affect litter decomposition in tropical forests and this impact may become stronger with forest succession in the studied tropical forests. Our results can provide relevant information on how future climate changes modify accumulation of organic matter (C) in tropical forest and consequently affect nutrient cycle, and for any sudden events in the forest such as hurricane and tree diseases which also changes quantity of litter input to the forest floor. However, recent mechanisms are still far from clear, thus we also suggest similar studies in temperate and other tropical forest ecosystems to further strengthen the findings.

## Supporting Information

Table S1 Indices of the tree structure in the three tropical forest types. The survey was conducted in February 2007.
(DOC)

Table S2 Initial chemical characteristics of three studied leaf litter.
(DOC)
