# Supplementary material for: Effects of Litter Manipulation on Litter Decomposition in a Successional Gradients of Tropical Forests in Southern China
Source: PLoS One. 2014 Jun 5;9(6):e99018. doi: 10.1371/journal.pone.0099018 (PMC4047082; doi:10.1371/journal.pone.0099018)
Supplement: Table S1 — Indices of the tree structure in the three tropical forest types. The survey was conducted in February 2007. (DOC) [file pone.0099018.s001.doc]

**Table S1.** Indices of the tree structure in the three tropical forest types. The survey was conducted in February 2007.

| Species | Stem  Density  (tree hm^-2^) | Mean  height  (m) | Mean of diameter breast  height  (cm) | Basal area  (m^2^ hm^-2^) | Percentage of basal area to total (%) |
| --- | --- | --- | --- | --- | --- |
| **MEBF** |  |  |  |  |  |
| Castanopsis chinensis | 268 | 9.8 | 26.0 | 18..7 | 49.0 |
| Machilus chinensis | 131 | 9.0 | 14.8 | 4.0 | 10.6 |
| Schima superba | 185 | 9.9 | 18.3 | 6.4 | 16.7 |
| Cryptocarya chinensis | 270 | 8.3 | 14.3 | 4.4 | 11.5 |
| Syzygium rehderianum | 185 | 8.5 | 12.9 | 1.2 | 3.1 |
| Other plants | 1587 | 4.3 | 4.4 | 3.5 | 9.1 |
| Total | 2625 |  |  | 38.2 | 100 |
| **MF** |  |  |  |  |  |
| Pinus massoniana | 240 | 9.1 | 20.5 | 9.9 | 38.9 |
| S.superba | 1600 | 3.9 | 4.2 | 4.2 | 16.5 |
| Other plants | 1307 | 4.3 | 7.6 | 11.3 | 44.6 |
| Total | 3147 |  |  | 25.4 | 100 |
| **MPF** |  |  |  |  |  |
| P.massoniana | 560 | 7.0 | 19.3 | 22.0 | 88.7 |
| Other plants | 1707 | 3.5 | 3.3 | 2.8 | 11.3 |
| Total | 2267 |  |  | 24.8 | 100 |
