# Supplementary material for: Effects of Litter Manipulation on Litter Decomposition in a Successional Gradients of Tropical Forests in Southern China
Source: PLoS One. 2014 Jun 5;9(6):e99018. doi: 10.1371/journal.pone.0099018 (PMC4047082; doi:10.1371/journal.pone.0099018)
Supplement: Table S2 — Initial chemical characteristics of three studied leaf litter. (DOC) [file pone.0099018.s002.doc]

**Table S2.** Initial chemical characteristics of three studied leaf litters

| Species | C (mg g^-1^) | N (mg g^-1^) | P (mg g^-1^) | C/N | C/P | N/P |
| --- | --- | --- | --- | --- | --- | --- |
| S. S. | 557 (5) b | 15.2 (0.4) b | 0.80 (0.01) a | 36.9 (1.2) a | 697 (12) c | 19 (0.7) c |
| C.C. | 537 (2) a | 13.2 (0.2) a | 0.84 (0.01) a | 40.6 (0.5) a | 643 (8) b | 15.9 (0.2) b |
| P. M. | 587 (3) c | 12.0 (0.5) a | 1.34 (0.02) b | 49.5 (2.4) b | 440 (8) a | 9 (0.5) a |
